# Numerical simulation of the influence of nasal cycle on nasal airflow

**DOI:** 10.1038/s41598-024-63024-9

**Published:** 2024-05-28

**Authors:** Jing Wei, Xuan He, Qing Yang, Qifei Gu, Xiaodan Zhang, Xue Sui, Rui Zhou, Wei Feng

**Affiliations:** 1 School of Chinese Materia Medica, Beijing University of Chinese Medicine, Beijing, China; 2 Department of Otolaryngology, Wangjing Hospital, China Academy of Chinese Medical Sciences, Beijing, China

**Keywords:** Nasal cycle, Computational fluid dynamics, Airflow characteristics, Numerical simulation, Nasal resistance, Pressure, Airflow velocity, Computational biology and bioinformatics, Medical research

## Abstract

To study the characteristics of nasal airflow in the presence of nasal cycle by computational fluid dynamics. CT scan data of a healthy Chinese individual was used to construct a three-dimensional model of the nasal cavity to be used as simulation domain. A sinusoidal airflow velocity is set at the nasal cavity entrance to reproduce the breathing pattern of a healthy human. There was a significant difference in the cross-sectional area between the two sides of the nasal cavity. Particularly, the decongested side is characterized by a larger cross-section area, and consequently, by a larger volume with respect to the congested side. The airflow velocity, pressure, and nasal resistance were higher on the congested narrow side. The temperature regulation ability on the congested narrow side was stronger than that on the decongested wider side. During the nasal cycle, there are differences in the nasal cavity function between the congested and decongested sides. Therefore, when evaluating the impact of various factors on nasal cavity function, the nasal cycle should be considered.

## Introduction

Kayser first discovered that the nasal mucosa of normal people is always in a state of alternating congestion and decongestion, which he named the “nasal cycle” in 1895^1^. Approximately 80% of normal people have this phenomenon^2^, which is manifested as changes in the size of the nasal turbinate on both sides. When one side of the nasal cavity is in a contracted state, the other side is in a relaxed state, varying every 0.5–7 h^3,4^. However, the turbinate regulates the inhaled air flow through the nasal cycle, so that the total volume and total resistance of the nasal cavity on both sides are maintained within a constant range. At different phases of the nasal cycle, the airflow inside the nasal cavity shows different patterns. The presence of the nasal cycle is a necessary condition to effectively regulate the respiratory airflow, and is also the reason why normal people do not feel nasal congestion.

Studies have shown that the nasal cavity functions corresponding to different phases of the nasal cycle are different to some extent^5^. Therefore, when evaluating the impact of various factors on nasal function, the influence of the nasal cycle should be fully considered. Various methods have been used to study the significance, mechanism and influencing factors of this physiological phenomenon. In the early years, Gotlib et al.^6^ used acoustic rhinometry to record the cross-sectional area of inferior turbinate in patients with rhinitis during 5–7 h of nasal cycle, and summarized the influence of nasal cycle on nasal stimulation test. Tahamiler et al.^7^ developed Odiosoft-Rhino software program to evaluate nasal airflow by spectrum and frequency analysis of nasal sounds, then objectively detect and evaluate nasal cycle. Rohrmeier et al.^8^ used a long-term rhinoflowmeter to measure the nasal airflow during wakefulness and sleep, compared the occurrence, duration and relative amplitude of nasal cycles during wakefulness and sleep, and studied the relationship between nasal cycles and body position. Previous studies have used techniques such as anterior active rhinomanometry (AAR) and peak nasal inspiratory flow (PNIF) to evaluate nasal cycles^9^. In recent years, computational fluid dynamics (CFD) has been gradually applied to nasal science research. CFD can simulate respiratory airflow patterns efficiently and reliably^10^. Patel et al.^11^ manually created a model based on computer CT scans before and after surgery to simulate various congestion states of nasal mucosa. By comparing the simulation results of airflow distribution and nasal resistance, the surgical changes of nasal patency can be distinguished from the physiological changes related to nasal circulation. Considering the constant suction flow rate of 250 mL/s, Byun et al.^12^ simulated the air conditioning characteristics of the nasal cycle using computational fluid dynamics. They found that there were significant differences in air temperature and relative humidity between the congested and decongested sides of the model in the presence of the nasal cycle. At present, most of the CFD research on the nasal cavity is aimed at studying the nasal cycle of patients with nasal congestion or rhinitis. This is a key factor in evaluating whether surgery for nasal cavity lesions is successful^13–15^. However, 80% of heathy people also have the nasal cycle. Study on nasal cycle is a preliminary study for the nasal delivery. The nasal cycle has an effect on airflow and volume in the nasal cavity. The flow rate and volume play an important role on the rate of drug deposition and position. Therefore, it is necessary to study the characteristics of the flow into the nasal cavity during the nasal cycle of healthy people. Compared with using instruments to study nasal temperature, pressure and nasal resistance, numerical simulation is more convenient and intuitive to reflect the instantaneous changes of internal flow field. Therefore, this study constructed a nasal cavity model based on medical CT data of a heathy person and used transient simulation to analyze the temperature, airflow velocity and pressure, and nasal resistance on both sides of the nasal cavity during the nasal cycle.

## Materials and methods

### Construction of the nasal cavity model

Our nasal cavity model is created from the CT scan data of a healthy male volunteer. This individual has no background of acute or chronic conditions related to the upper respiratory tract, maintains a regular body mass index, and has no previous experiences of trauma or surgery in the maxillofacial and upper respiratory areas. The study received approval from the Medical Ethics Committee of Beijing University of Traditional Chinese Medicine (Approval Number: 2023BZYLL1018). The volunteer has also given his informed consent for this clinical research. All methods were performed in accordance with the relevant guidelines and regulations. The scanned CT images were imported into 3D reconstruction software Mimics and Geomagic Studio for processing. The nasal cavity airway model was simplified by removing the anatomical structures of the maxilla, ethmoid sinuses, sphenoid sinuses, and frontal sinuses, which have a small effect on nasal airflow^16^. The final smooth surface was generated and imported into the ANSYS platform for numerical simulation.

**Figure 1:**
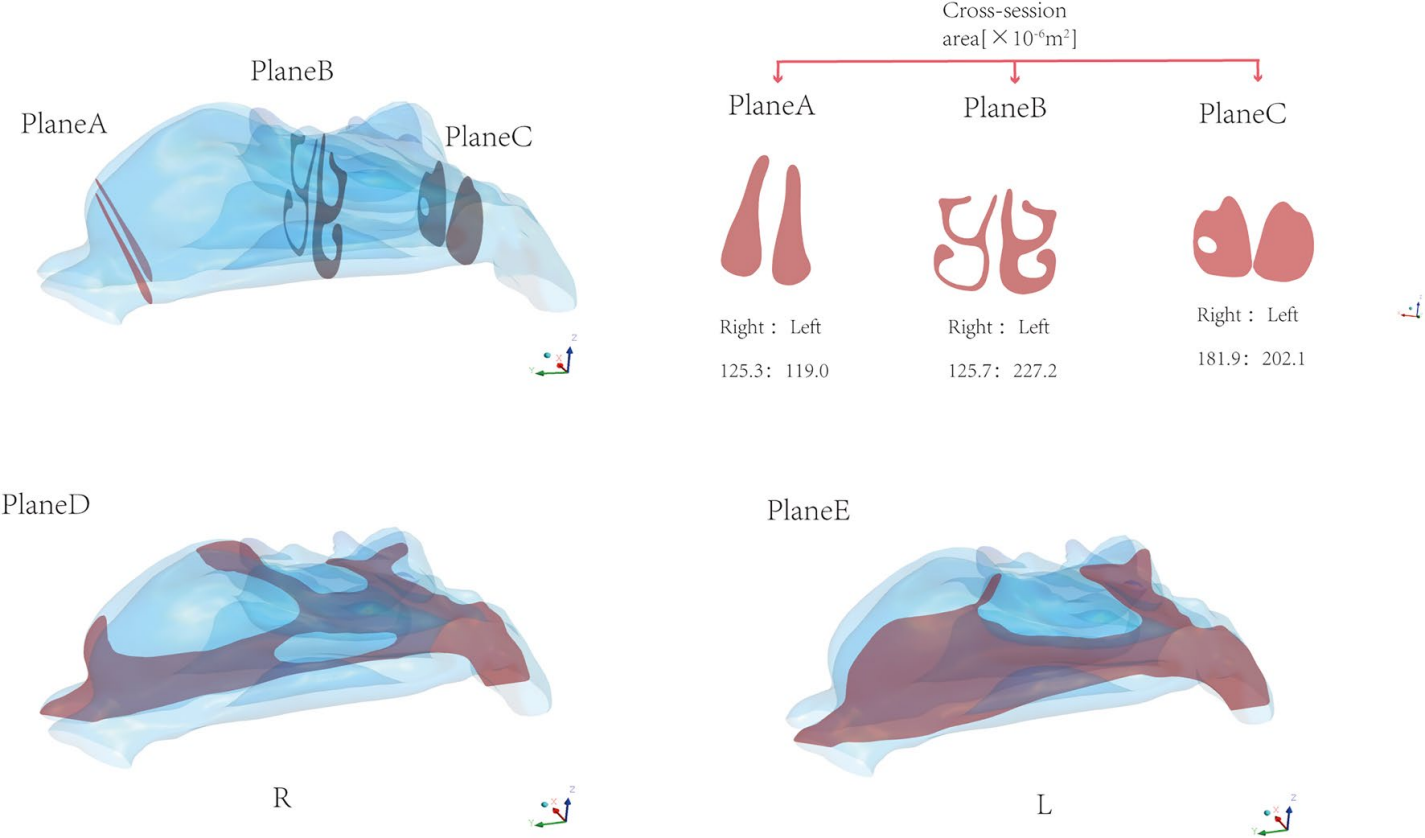
Representative planes: Plane A, Plane B, and Plane C are representative planes perpendicular to the gas flow direction. Plane D and Plane E are sagittal planes on the right and left sides of the nasal cavity.

To present the simulation results, three representative two-dimensional planes were selected for examining the local flow field, as shown in Fig. 1. The selected planes were roughly perpendicular to the direction of gas flow to study the changes in the flow field and pressure field along the actual streamline more effectively. The distance from the airway to the nostril was normalized by the length of the nasal septum. The first plane (Plane A) represents the entrance position, and the last plane (Plane C) represents the end position of the nasal septum. Plane B is located in the middle of the airway and reflects the airflow characteristics of the middle part of the airway. Planes D and E were also established as sagittal planes to reflect the airflow characteristics on both sides of the nasal cavity.

### Mesh generation

**Figure 2:**
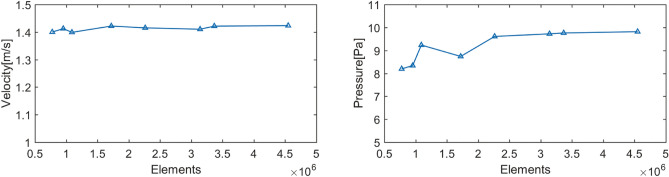
Variation of outlet velocity and total pressure drop in the airway with different number of elements in the mesh.

**Figure 3:**
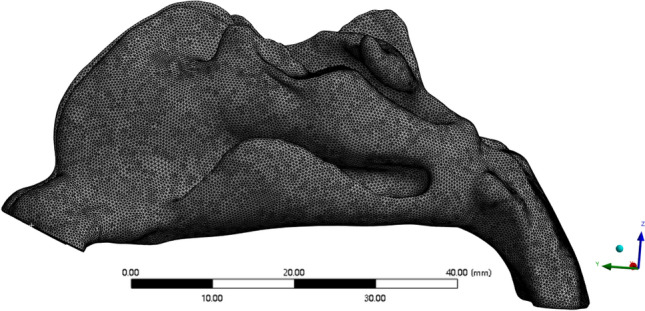
Schematic diagram of the mesh.

The meshing was performed in ANSYS Mesh, and the upper respiratory tract outlet, inlet, and wall were defined. Before the formal calculation, mesh independence verification was performed. Eight meshes with different level of refinement were used. The number of nodes and elements is shown in Table 1. The pressure drop along the nasal cavity and outlet velocity at the maximum inhalation time t = 1.25 s were calculated to verify the independence of the calculation results on the mesh. Figure 2 shows the variation of pressure drop and outlet velocity with the number of mesh elements. From the Fig. 2, it can be seen that the outlet velocity changes slightly with the number of elements, whereas the pressure drop varies significantly. When the number of elements is sufficiently high, the pressure drop tends to be stable. It can be seen that the calculated results of Mesh 5 and Mesh 6 are very similar. The relative error of the pressure drop between Mesh 5 and Mesh 6 is 1.1%. Therefore, it can be considered that the flow field obtained by numerical simulation is relatively stable when the total number of elements exceeds 2.26 million. Considering the factors of calculation accuracy and efficiency, the mesh with a total number of nodes of 677,356 and elements of 2,262,838 was used in the subsequent calculations. The successfully divided mesh is shown as in the Fig. 3.

**Table 1:** Number of nodes and elements after domain discretization.

	Mesh 1	Mesh 2	Mesh 3	Mesh 4	Mesh 5	Mesh 6	Mesh 7	Mesh 8
Nodes	253,259	305,661	349,700	528,308	677,356	917,193	972,860	1,282,572
Elements	776,909	953,073	1,091,424	1,720,761	2,262,838	3,140,611	3,367,343	4,548,892

### Numerical simulation settings

ANSYS Fluent 19.2 was used for computational fluid dynamics (CFD) analysis. In the CFD calculations, nasal airflow is considered to be viscous, turbulent and incompressible and the fluid Newtonian Reynolds Averaged Navier–Stokes equations are used in this study to simulate the nasal airflow. The k-ε model is used to close the system of equations. The SIMPLE algorithm was chosen to solve the equations numerically.

**Figure 4:**
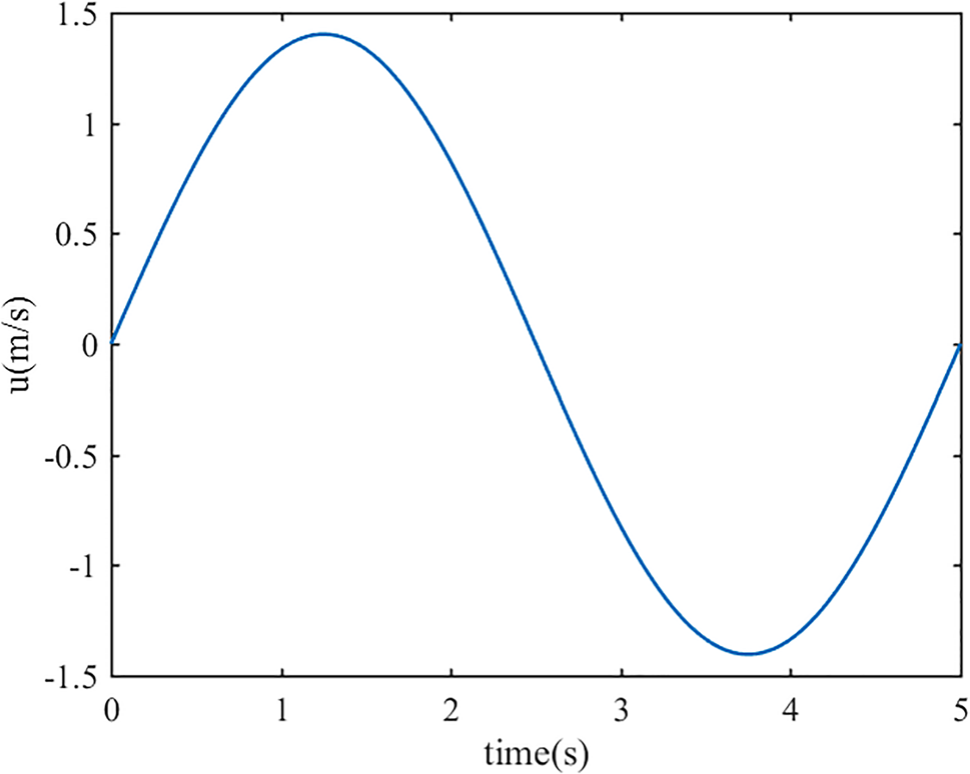
The variation of inlet velocity with time for a complete respiratory cycle.

A “velocity-inlet” boundary type is set at the nasal cavity entrance. A User Defined Function was written to impose a sinusoidal velocity value (Fig. 4), with a respiratory period T = 5 s and an inspiratory-to-expiratory ratio of 1:1, simulating the breathing pattern of a heathy human at rest with an inspiratory flow rate of 15 L/min. The inhaled air is assumed to be at typical atmospheric conditions of 25 °C^17^. The outlet is set to be a pressure outlet (P = 0) and the temperature at the outlet is assumed to be 36 °C, close to alveolar conditions^18^. The wall of the nasal cavity is rigid and stationary and a no-slip condition is set at this boundary. The temperature of the wall is set as 36 °C.

## Results

### Geometrical characteristics of the nasal cavity model

**Figure 5:**
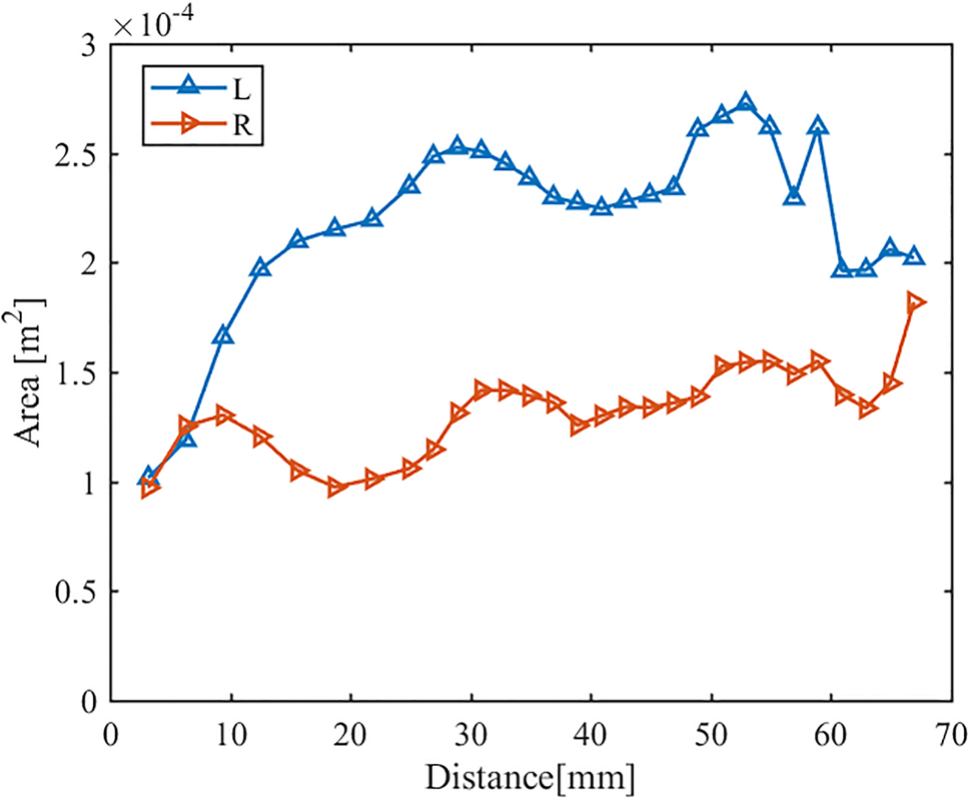
Cross-sectional area distribution perpendicular to the local flow direction.

In Fig. 5, we can see the changes in cross-sectional area of the airway from the nostrils to the end of the nasal septum. While previous studies used nasal coronal plane information to reflect changes in cross-sectional area, it is worth noting that the overall flow direction of the airflow at the front end of the nasal cavity tends to be inclined to the coronal plane. The plane obtained along the flow direction can more accurately describe the physical information of the flow field. Jo et al. showed that there is a significant difference in the area of the obtained plane at the nasal valve^19^. Therefore, the plane selected here is roughly perpendicular to the local flow direction. We selected 29 planes from the nostrils to the end of the nasal septum to obtain more continuous flow field information.

The cross-sectional area of the left (L) and right (R) nasal passages shows that the left side is the non-congested side, and the cross-sectional area of the airway is much larger than that of the congested right side. Table 2 summarizes the nasal airway volume, surface area of the nasal cavity wall, and surface area-to-volume ratio (SAVR). Although the volume of the decongested side of the model is much larger than that of the congested side, it is found that the difference in surface area is small, and even the surface area of the non-congested left side is smaller than that of the congested right side. Byun et al. also found this phenomenon in their study^12^. Therefore, the existence of nasal cycle may not have a significant impact on surface area, but mainly on changes in airway volume. As a result, the SAVR on the congested side is much larger than that on the non-congested side.

**Table 2:** Geometric Characteristics of the Model.

	Left	Right
Volume of the airway from the nostrils to the end of the nasal septum/10^3^ mm^3^	14.456	8.898
Surface area of airway from nostrils to the end of nasal septum/10^3^ mm^2^	7.680	8.015
Surface area to volume ratio/mm^−1^	0.531	0.901

### Pressure and nasal resistance

**Figure 6:**
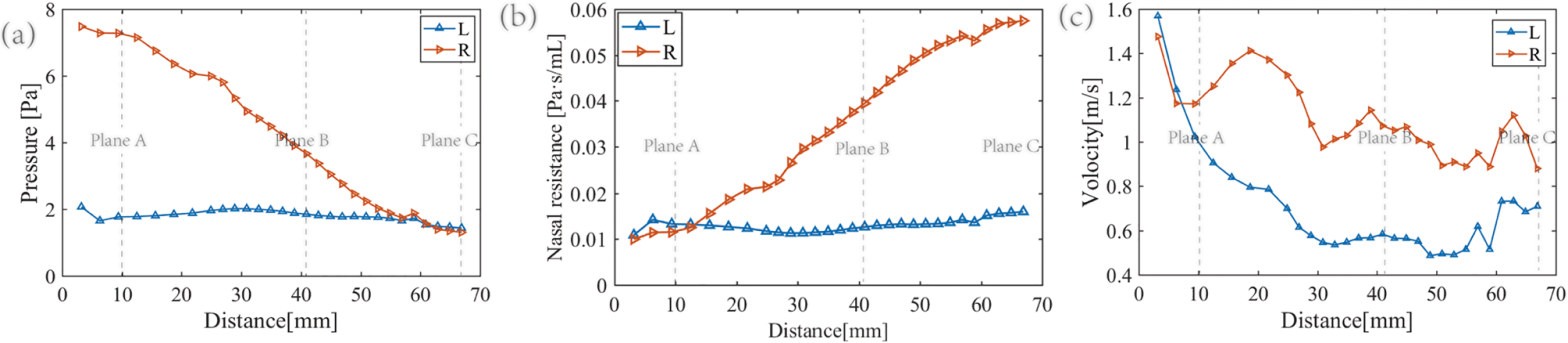
(**a**) Represents the average pressure at different cross-sections from the entrance to the end of the nasal septum at t = 1.25 s. (**b**) Represents the nasal resistance at different cross-sections from the entrance to the end of the nasal septum at t = 1.25 s. (**c**)  Represents the airflow velocity at different cross-sections from the entrance to the end of the nasal septum at t = 1.25 s.

**Figure 7:**
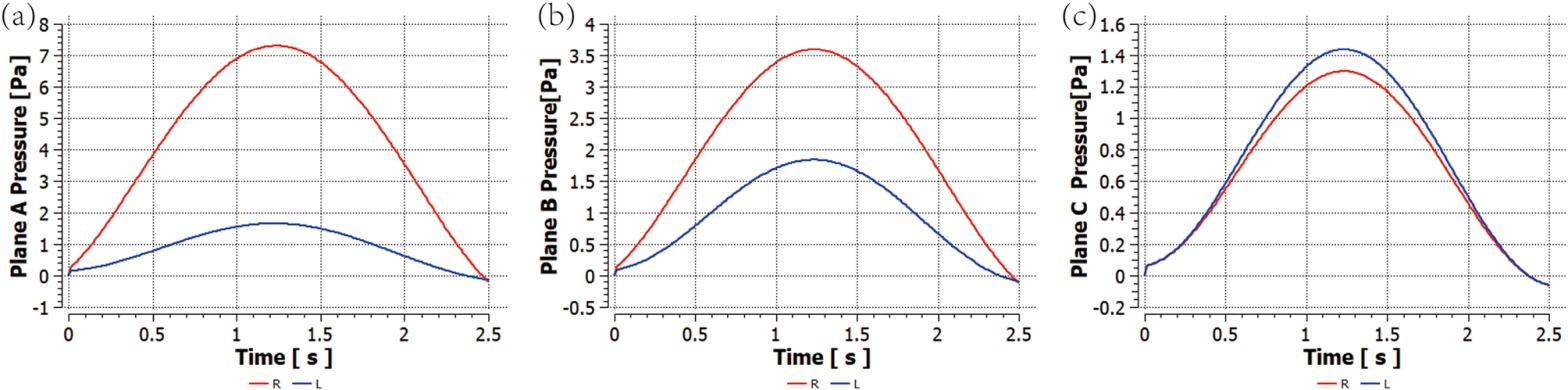
(**a**) Represents the pressure change of Plane A with time. (**b**) Represents the pressure change of Plane B with time. (**c**) Represents the pressure change of Plane C with time.

In Fig. 6(a), pressure is shown at different cross-sections along the nasal cavity, from the nasal entrance to the end of the nasal septum during the moment of maximal inhalation (t = 1.25 s) in the breathing cycle. The pressure in both left and right nasal cavities decreases along the direction of airflow, with the pressure in the congested right nasal cavity decreasing more significantly in a linear trend. The pressure in the left nasal cavity decreases less noticeably and remains relatively stable. Figure 7 shows the pressure changes over time on three representative planes in both left and right nasal cavities. The pressure on all three planes shows a roughly sinusoidal trend over time, as determined by the inlet boundary condition. The transient pressure on the right side consistently remains higher than that on the left side. Plane A, near the entrance, has the highest pressure overall among the three planes, while Plane C, near the exit, has the lowest pressure. These trends are consistent with the pressure variations along the nasal cavity at t = 1.25 s shown in Fig. 6(a). In fact, the pressure decreases along the direction of airflow, so that the pressure difference between the left and right sides becomes smaller and the pressures on both sides is almost equal at Plane C.

Figure 6b also shows the changes in unilateral nasal resistance along the direction of airflow at t = 1.25 s. Nasal resistance is defined as ΔP/Q, where ΔP is the pressure difference between the specified plane and the entrance plane, and Q is the flow rate^20^. Note that, according to its definition, nasal resistance and pressure show an opposite trend. The former increases along the direction of the airflow while the latter decreases. On the congested right side, the total nasal resistance is 0.0575 Pa s mL^−1^, while on the non-congested left side, it is 0.0160 Pa s mL^−1^. Their ratio is 3.60, which is within the range reported in previous studies13. The slighter pressure reduction—and therefore, slighter nasal resistance—along the nasal cavity in its left side could be interpreted as smaller pressure loss due to the larger cross-section area.

### Distribution of airflow velocity

**Figure 8:**
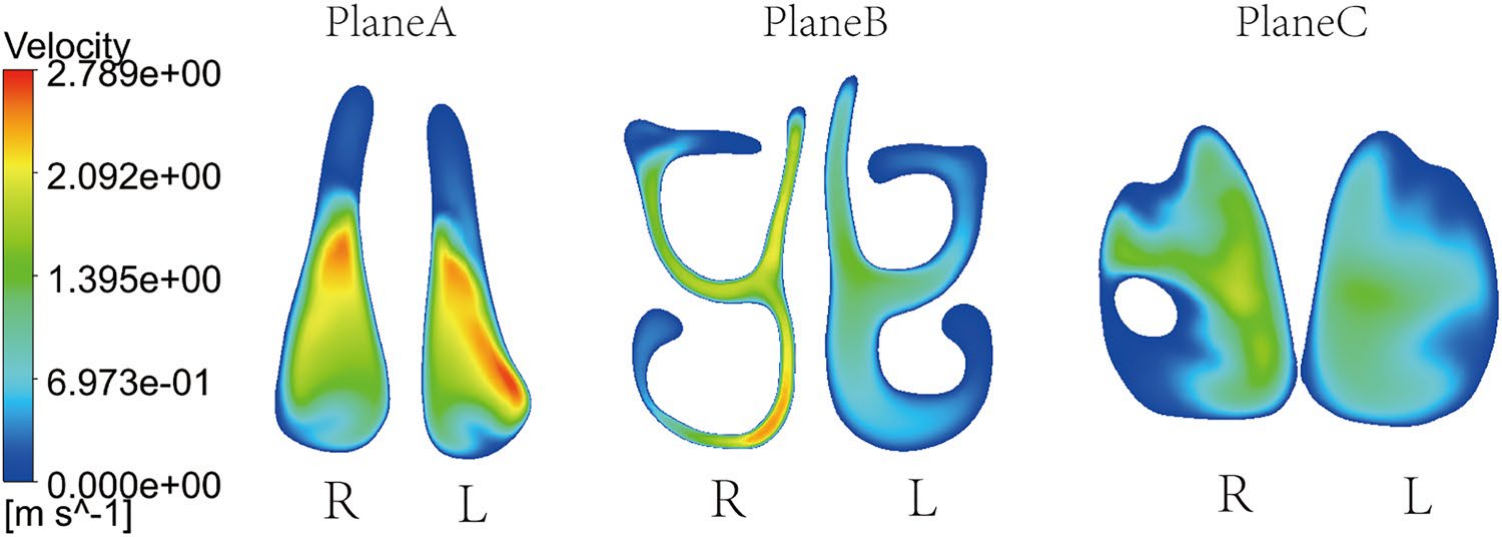
Velocity cloud maps of representative planes Plane A, Plane B, and Plane C simulated at t = 1.25 s.

The average value of velocity within different cross-sections of the nasal cavity is shown in Fig. 6c. Note that, provided a given airflow rate flowing through one side of the nasal cavity and according to mass conservation, the airflow velocity and cross-section area have opposite trend. The airflow velocity increases (decreases) as the cross-section area decreases (increases). Also, note that almost the same airflow rate enters the two sides of the nasal cavity, as the same inlet velocity is set and the inlet cross-section area is almost the same. Therefore, the smallest cross-section area in the congested side results in an overall higher airflow velocity. In Fig. 8 the spatial distribution of the airflow velocity within the three different cross-sections, Plane A, Plane B and Plane C is shown. From the velocity cloud chart (Fig. 8), the velocity difference between the left and right sides of plane A is not significant. The maximum velocity on the left side is located in the middle and lower parts of the nasal cavity. As the airflow progresses, the difference in area between the left and right sides of the nasal cavity becomes more apparent, and the velocity difference becomes more pronounced at Plane B. The flow velocity on the right nasal passage is higher with respect to the left side at Plane C as well. As already pointed out before, overall, the air velocity on the right nasal passage is higher than that on the left nasal passage. Besides, the flow velocity at the three sections shows a decreasing trend from the centre to the edge of the airway.

### Temperature field distribution

**Figure 9:**
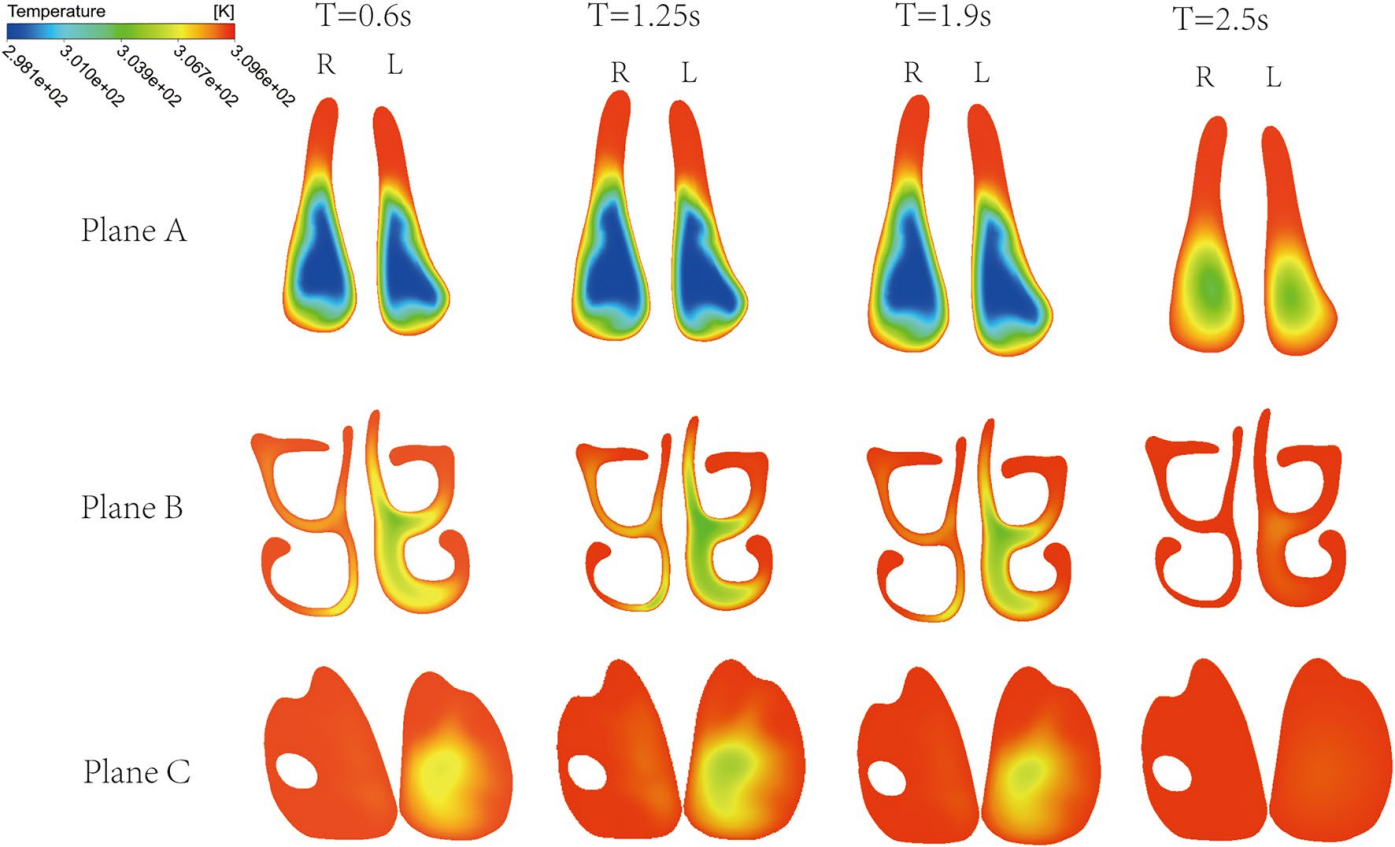
Instantaneous temperature cloud maps of Plane A, Plane B, and Plane C.

The underside of the nasal mucosa and septum are covered with numerous capillaries, which regulate the temperature of gases entering the respiratory system through them. The temperature spatial distribution within Plane A, B and C at different time instant during the inhalation phase is shown in Fig. 9. At Plane A the temperature is on average lower with respect to the other planes during the whole inhalation duration. This is due to the fact that plane A is closer to the nostril, where fresh ambient air enters the nasal cavity. In fact, the temperature of the nasal cavity wall is set at a higher value (36 °C) with respect to the entering fresh air (25 °C). Therefore, heat exchange occurs between the walls and the air into the nasal cavity and the air temperature globally increases along the airflow direction, i.e. from Plane A to Plane C. Comparing the two sides of the nasal cavity, the average temperature within the cross-section is lower in the decongested side (see e.g. left and right sides of Planes B and C). However, at the entrance of the nasal cavity (Plane A) the temperature is similar in the two sides. Looking at the time variation of the temperature into the nasal cavity, it seems that at the end of the inhalation phase (t = 2.5 s), when the inlet airflow velocity is low, the temperature into the nasal cavity is overall higher with respect to previous time instants. Particularly, at Plane C the air has almost the same temperature as the nasal cavity walls. Note that the temperature is not uniform within the cross-section, but it is higher the closer to the walls.

**Figure 10:**
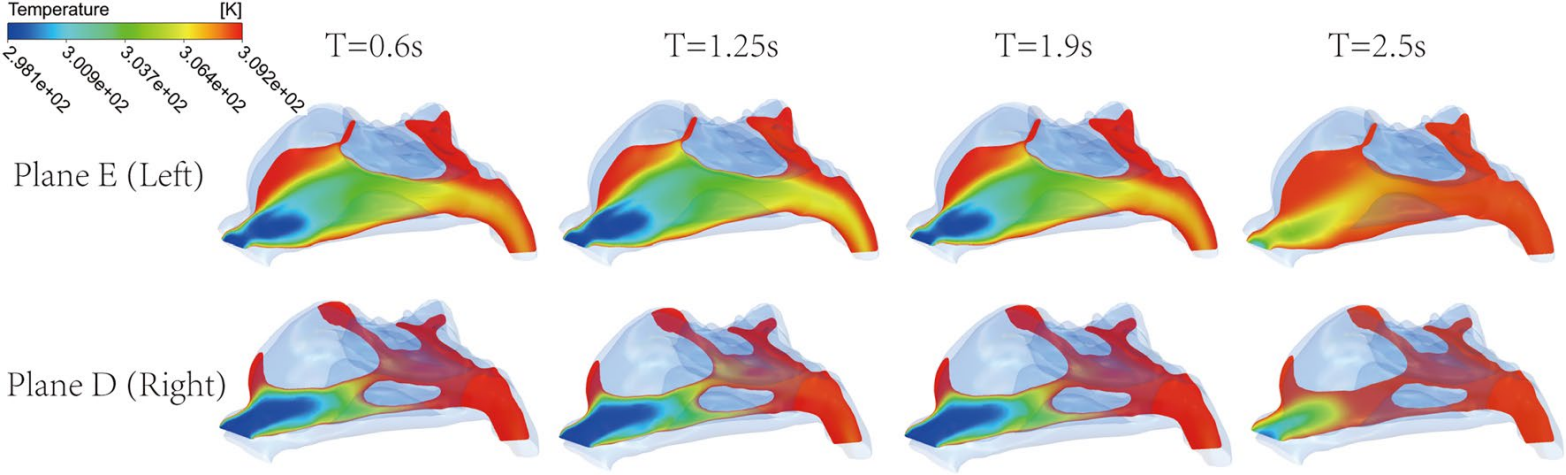
Instantaneous temperature cloud maps of Plane D and Plane E.

The temperature cloud maps of Plane E and Plane D, which represent the left and right nasal cavities respectively, change over time, as shown in Fig. 10. The temperature at the inlet was set at 25 °C, and the wall and outlet temperatures were set at 36 °C. Therefore, the temperature of the airflow is lowest at the inlet and increases rapidly due to heat exchange in the nasal cavity, finally reaching the temperature of the wall and outlet. Comparison of the two sides shows that the surface temperature of the congested side at the rear of the nasal cavity is higher. Generally speaking, in the congested right side the air reaches the temperature of the nasal cavity wall at a shorter distance from the nostril. Particularly, the air temperature has already reached the wall temperature in the middle of the nasal cavity in the right side, whereas in the left side the air temperature is still below 36 °C at the outlet boundary in the time interval 0.6 s ~ 1.9 s.

**Figure 11:**
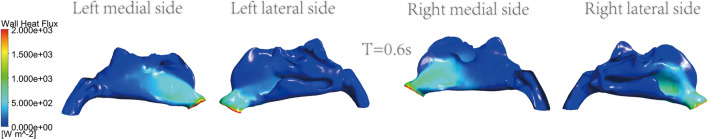
Instantaneous cloud maps of heat exchange on the medial and lateral sides of both nasal cavities at T = 0.6.

**Figure 12:**
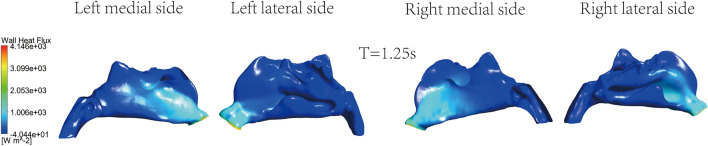
Instantaneous cloud maps of heat exchange on the medial and lateral sides of both nasal cavities at T = 1.25.

**Figure 13:**
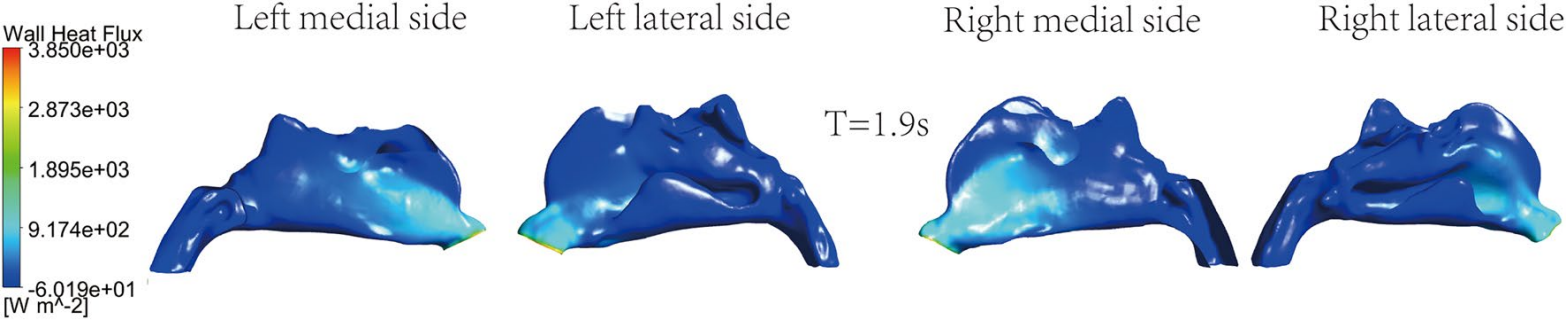
Instantaneous cloud maps of heat exchange on the medial and lateral sides of both nasal cavities at T = 1.9.

**Figure 14:**
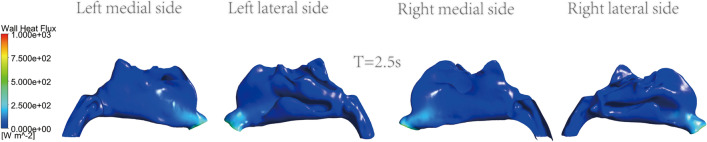
Instantaneous cloud maps of heat exchange on the medial and lateral sides of both nasal cavities at T = 2.5.

The instant cloud maps of the medial and lateral heat exchange of the left and right nasal cavities are shown in Figs. 11, 12, 13 and 14. The distribution of surface heat flux is similar to that of temperature. Heat exchange is significant at the entrance of the nasal cavity, but there is almost no heat exchange in the middle and rear sections of the nasal cavity. The heat flux of the right nasal cavity is slightly higher than that of the left. The heat flux on the airway surface changes with time, showing an increasing and then decreasing trend. The heat flux is the largest at T = 1.25 s, as shown in Fig. 12. The entrance is light blue at the end of inspiration, which means the less heat exchange here, as shown in Fig. 14.

**Figure 15:**
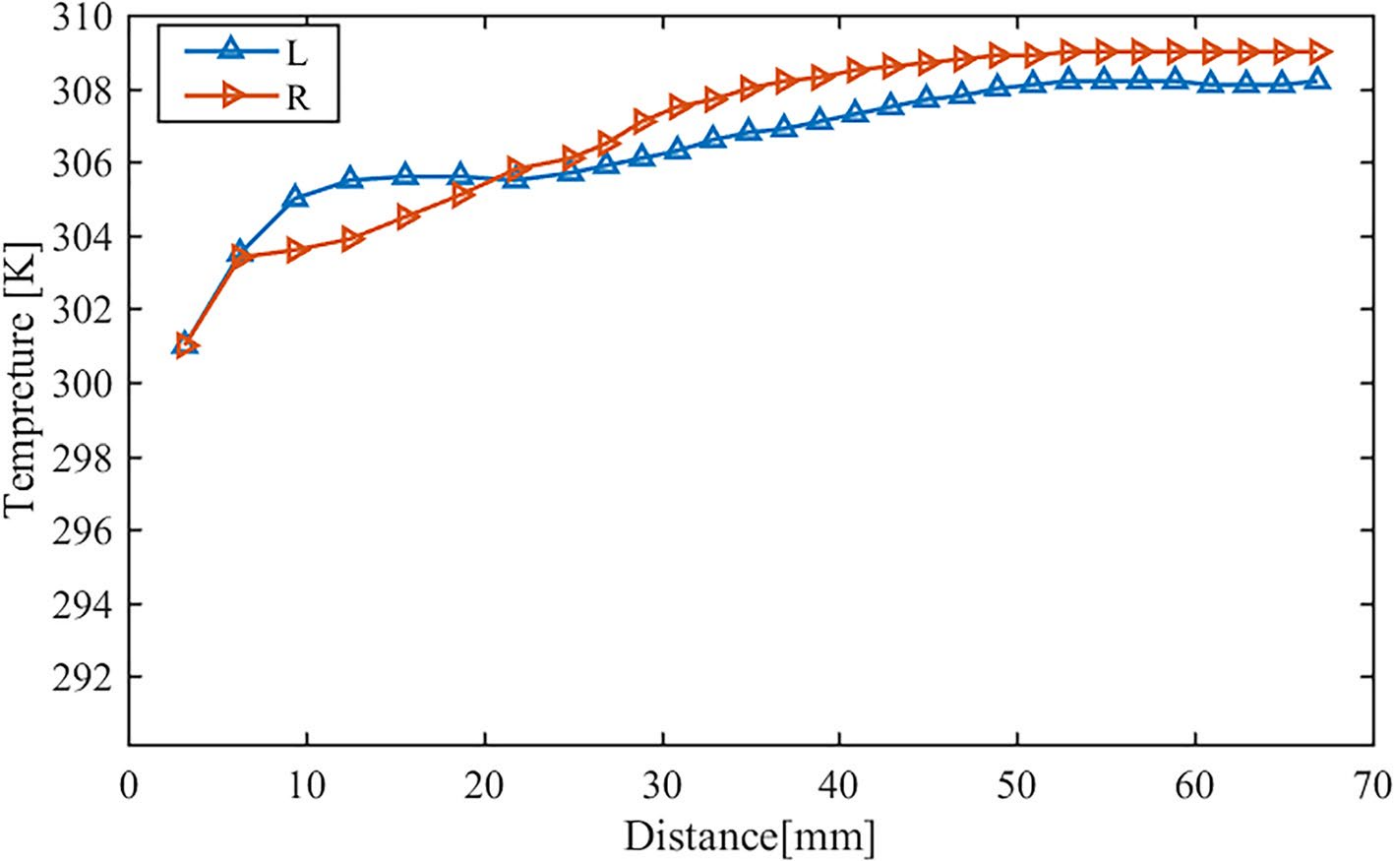
Distribution of temperature of inhaled air at the end of inspiration along the distance from the nostrils.

The change in the temperature of inhaled air with position at t = 1.25 s is shown in Fig. 15. The temperature is not homogeneous in the cross-section. Therefore, to analyse the temperature evolution along the nasal cavity, the average value in the cross-section is considered. It is found that the air temperature from the anterior 20 mm of the nasal cavity to the end of the nasal septum is higher on the congested side. In the front and middle parts of the left and right nasal cavities, the temperature curve has a large slope and the temperature rises rapidly as the gas inlet from the entrance of the nasal cavity. This area corresponds to the inferior turbinate area of the nasal cavity. The heat exchange between the wall and the gas is higher in the inferior turbinate area. The temperature difference between the nasal cavity wall and the gas is large, and the temperature gradient in the flow field is large. The adjustment of heat in the nasal cavity to the gas is mainly achieved by the heat transfer of the wall. After entering the nasal cavity, the gas exchanges heat with the wall the nasal cavity and the gas is slowly heated up. When the airflow reaches the middle and posterior parts of the nasal cavity, the slope of the gas temperature curve decreases significantly, and the temperature tends to be stable. Especially in the right nasal cavity, the temperature of the gas (about 309 K) almost identical with the temperature of the wall (309.15 K).

## Discussion

The nasal airway structure data of this model was obtained from a healthy male with no apparent anatomical variations in the nasal cavity. The model was used to investigate the air conditioning characteristics during the inhalation part of one breathing cycle.

The nasal cycle is characterized by the alternating contraction and relaxation of the left and right nasal cavity mucosa. This causes alternating changes in nasal airway volume and resistance, which regulate the primary ventilation function of each side of the nasal cavity. Nasal resistance is lower on the side with a larger cross-sectional area. The alternating primary ventilation function of the bilateral nasal cavities relieves the alternating breathing load of both sides and can protect the nasal cavity, especially the upper and lower parts of the total nasal cavity. Another major physiological function of the nasal cycle is to provide necessary temperature regulation for the inhaled air, making it similar to the conditions of the alveoli in the nasopharynx in terms of temperature, humidity, etc. During inhalation, the airflow velocity changes rapidly, and the surface's transient response to these changes may be critical for nasal physiology based on thermal capacity evaluation. The highest values of heat flux were found on the anterior side of the nasal cavity, indicating a significant heating effect on the airflow. Consequently, the air temperature increases rapidly in this zone of the nasal cavity. After passing through the nasal valve area, the airflow velocity decreases, extending the time of contact between the airflow and mucosa, which is also conducive to the temperature regulation of the mucosa for the gas.

The article examines the characteristics of the nasal cycle by analysing differences in nasal cross-sectional area, airflow velocity, airflow pressure, nasal resistance, and temperature regulation ability. The results of the present work suggest that the congested side of the nasal cavity is not completely dormant but exhibits some activity. In fact, it is observed that some air flows through the congested side. This study has certain limitations that should be acknowledged. Future research should focus on the duration of the nasal cycle and its performance at different times. It is also essential to note that this article only considers the inspiration state of the nasal cycle and assumes that expiration is the opposite process of inspiration. In reality, these two behaviours are not entirely identical, and future studies must analyse the entire respiratory cycle. Moreover, improving the wall model is crucial, as the real nasal cavity wall involves complex heat transfer processes. Although this study sets a simple wall model that reflects the characteristics of nasal temperature regulation, there is always room for improvement.

The typical expression of the nasal cycle is the alternating congestion and decongestion of the nasal mucosa on the left and right sides. However, in fact, there are various types of nasal cycles, which have been classified in the literature. Most subjects exhibit one or several types of these cycles^21,22^. Due to a large amount of computational cost, only one typical nasal cycle model was analysed in this study. In future studies, multiple types of nasal cycles need to be considered to elucidate more accurate nasal cycle laws.

Lastly, validation of the CFD simulations against experimental data should be carried out, even if complex.

## Conclusion

Normal human nasal mucosa is always in the alternating changes of congestion and decongestion. The cycle is rotated every 2–7 h. However, due to the influence of body position, the nasal cycle may change in a short time. This paper discusses the characteristics of airflow changes into the nasal cavity when the volume of its left and right sides changes due to the nasal cycle. A nasal cavity model showing alternating nasal cycle states was simulated using computational fluid dynamics. A sinusoidal velocity inlet was used, and pressure and temperature fields were obtained in addition to the velocity field. The results showed significant differences in temperature, pressure, and nasal resistance between the congested and non-congested sides of the nasal cavity in the presence of the nasal cycle. Airflow velocity, pressure, temperature, and nasal resistance were generally higher in the congestive side cavity than in the decongestive side. When evaluating the influence of various factors on nasal cavity function, the nasal cycle should be fully considered.

The method presented in the present paper to model the airflow into a specific patient’s nasal cavity can be useful for several applications. Building a model of the patient’s nasal cavity before a surgery can be used to evaluate the best surgical plan. Besides, surgical outcome can be assessed by comparing the nasal cavity model before and after surgery. Further developments of the work include the study the intranasal flow field under different conditions of respiratory intensity in subsequent studies, including mild respiration in shock, normal respiratory pattern in calm state, and heavy respiration in acute respiratory state. In addition, the nasal cycle discussed in this study has a significant effect on the velocity of the airflow and volume of the nasal cavity. Velocity of airflow and volume of the nasal cavity play an important role in the rate and location of drug deposition. Therefore, the study of the nasal cycle is essential as a basic study of nasal drug delivery. In future works, we will utilize the basis of this research to further investigate nasal drug delivery.

## Data Availability

The datasets generated during and/or analysed during the current study are available from the corresponding author on reasonable request.
